# Nitrogen-modulated intercropping boosts yield and quality in *Codonopsis pilosula*

**DOI:** 10.1186/s12870-026-08835-w

**Published:** 2026-04-28

**Authors:** Yi Xing, Minli Huang, Huifeng Miao, Qiong Wang, Qiang Zhang, Zhiping Yang, Gaojian Huang

**Affiliations:** 1 Soil Health Laboratory in Shanxi Province, College of Resource and Environment, Shanxi Agricultural University, Jinzhong, 030801 China; 2 Institute of Eco-Environment and Industrial Technology, Shanxi Agricultural University, Taiyuan, 030031 China; 3 Soil Health Laboratory in Shanxi Province, Taiyuan, 030031 China

**Keywords:** *Codonopsis pilosula*, Intercropping, Nitrogen, Photosynthesis, Carbon metabolism, Medicinal quality

## Abstract

Intensive monoculture of *Codonopsis pilosula* (*C. pilosula*) reliant on high nitrogen (N) inputs often compromises root quality and long-term sustainability. Here we show that intercropping with faba bean under optimized N application resolves this trade-off by enhancing root yield and the accumulation of active ingredients. To test this, we established a two-factor factorial design with monoculture vs. intercropping across three N rates (0, 60, 120 kg N ha⁻^1^). Root yield, concentrations of three key active ingredients (lobetyolin, atractylenolide III, and syringin), leaf traits, photosynthetic parameters, and the activities of key carbon and nitrogen metabolic enzyme were measured. Intercropping increased root yield by 3.2%-18.7%, active ingredient concentrations by 5.5%-56.9%, and active ingredients yield by 29.7%-47.1%. Mechanistically, these improvements were associated with key physiological changes: 1) enhanced photosynthetic capacity (larger leaves with higher photosynthetic rate) increasing total carbon assimilation; 2) upregulated C/N metabolism strengthening utilization and storage capacity of photosynthetic assimilates in root. These benefits were achieved despite a decrease in instantaneous water use efficiency, a pattern consistent with a “water-for-carbon” strategy at the leaf level. The intercropping advantage for *C. pilosula* was maximized at 60 kg N ha^−1^, while excessive N application (120 kg N ha^−1^) diminished these benefits and reduced active ingredient concentrations. Our findings demonstrate that faba bean intercropping under moderate N promotes both productivity and medicinal quality of *C. pilosula* by co-optimizing leaf physiology and C/N metabolism, providing a potential strategy for more sustainable cultivation under the conditions tested.

## Introduction

*Codonopsis pilosula* (*C. pilosula*) is a perennial medicinal herbaceous plant widely distributed in East Asia, with its primary suitable ecological region concentrated in China, covering approximately 884.79** × **10^4^ km^2^ of potential cultivation area globally [1]. Within China, the main production areas are located in Shanxi, Gansu, Shaanxi, and Sichuan provinces, where the unique climatic and soil conditions favor high-quality root production [1]. The root is used in traditional Chinese medicine and contains a diverse array of pharmacologically active compounds, including polysaccharides, lobetyolin (a polyacetylene glycoside), atractylenolide III (a sesquiterpene lactone), and syringin (a phenylpropanoid glycoside) [2]. These active ingredients possess both medicinal and nutritional value. In traditional Chinese medicine, *C. pilosula* is recognized for its effects on nourishing the middle (strengthening the digestive system) and supplementing qi (vital energy), as well as strengthening the spleen and lungs-functions that support overall vitality and immune health [3]. Due to the scarcity of wild resources and its specific growth environment requirements, *C. pilosula* is often continuously cultivated on the same land, leading to severe continuous cropping obstacles characterized by declining yield and active ingredient content [4]. Moreover, high fertilizer inputs, particularly nitrogen (N), are commonly applied to maximize root yield. This practice not only risks environmental pollution through leaching and volatilization but can also disrupt the balance between vegetative growth and secondary metabolism, potentially reducing the content of active ingredients [5, 6]. Therefore, there is a critical need to develop sustainable agronomic practices that can simultaneously enhance both root yield and medicinal quality of *C. pilosula*.

Intercropping with legumes has emerged as a promising strategy to enhance resource use efficiency and system productivity [7, 8]. Previous studies have documented yield improvements in various medicinal species under intercropping, including *Platycodon grandiflorus* with *Allium fistulosum* and *Panax notoginseng* cultivated under certain tree species [9, 10]. However, the mechanisms underlying these improvements have primarily been attributed to changes in soil microecology and nutrient availability [9, 11–13], leaving a critical knowledge gap: how does intercropping modulate the source-organ (leaf) to drive improvements in sink-organ (root) growth and secondary metabolism? A comprehensive understanding of this “source-to-sink carbon allocation regulation” is still lacking for *C. pilosula*-based systems.

Understanding “source-to-sink” regulation is essential because root yield and secondary metabolite accumulation are closely tied to the supply of photoassimilates from leaves. Leaf morphological traits, such as area and specific leaf area, determine light interception capacity. Physiological traits, including photosynthetic rate and stomatal behavior, govern carbon (C) assimilation efficiency [14]. Moreover, the activities of key C and N metabolic enzymes – such as sucrose synthase (SS), nitrate reductase (NR), and glutamine synthase (GOGAT)—serve as crucial hubs governing the conversion, transport, and partitioning of assimilates [15]. Despite their importance, systematic evaluations of these leaf-level physiological responses in intercropped medicinal plants remain scarce.

Nitrogen management is a pivotal factor modulating interspecific interactions in legume-based intercropping. While moderate N application can promote plant growth, excessive N suppresses legume N_2_ fixation, weakening the complementary N facilitation for the companion crop [16]. Furthermore, higher N availability often stimulates competitive aboveground growth, intensifying light competition and potentially reducing resource allocation to the roots and secondary metabolism [17]. Recent advances in intercropping research have demonstrated that optimized planting patterns and management practices can enhance system productivity by minimizing interspecific competition [18, 19]. However, the N-dependent modulation of interspecific interactions in *C. pilosula*/faba bean intercropping systems, and its consequences for source-sink carbon allocation remains unexplored.

To address these knowledge gaps, we conducted a field experiment with a factorial design incorporating two planting patterns and three N application rates and tested three hypotheses: (1) intercropping with faba bean would increase root yield and active ingredient content of *C. pilosula* compared to monoculture; (2) these improvements would be associated with enhanced leaf functional traits, photosynthetic performance, and the activity of key carbon and N metabolic enzymes; and (3) the yield advantages of the intercropping system would be optimized under an appropriate, rather than excessive, N application rate. By examining the underlying agronomic and physiological responses, this research aims to contribute to the scientific understanding of physiological mechanisms underlying intercropping responses in *C. pilosula*, which may inform the development of more sustainable cultivation.

## Materials and methods

### Site description and experimental design

The experiment was conducted in 2022 at the medicinal herb cultivation base of Shanxi Agricultural University in Lingchuan County, Shanxi Province, China (35°48′N, 113°27′E). This region features a typical temperate continental climate, with an elevation of 1200 m, an average annual rainfall of 650 mm, and an average annual temperature of 7.9 °C. The soil at the experimental site is typical cinnamon soil, with the following pre-experiment measurements (0–20 cm depth): organic matter 28.72 g kg⁻^1^, total N 2.06 g kg⁻^1^, available N 32.80 mg kg⁻^1^, available phosphorus (P) 13.99 mg kg⁻^1^, available potassium (K) 206.55 mg kg⁻^1^, and a pH of 8.13. This area is a genuine production region for *C. pilosula* (Lu Dangshen).

The experiment adopted a two-factor randomized block design. The first factor consisted of three N application levels: no N (N0), 60 kg N ha⁻^1^ (N60), and 120 kg N ha⁻^1^ (N120). Half of the N (urea) was applied as basal fertilizer before sowing, along with 40 kg P ha⁻^1^ (calcium superphosphate) and 45 kg K ha⁻^1^ (potassium sulfate). The remaining N was top-dressed at the initial flowering stage of *C. pilosula*. The second factor included two cropping systems: monocropped *C. pilosula* and intercropping of *C. pilosula* with faba bean (*Vicia faba*), resulting in a total of six treatments. Each treatment was replicated four times.

*Codonopsis pilosula* (Franch.) Nannf. seedlings (variety: ‘Ludangshen’) were sourced from the medicinal plant nursery of Shanxi Agricultural University in Lingchuan County, Shanxi Province, China. The plant was identified as ‘Ludangshen’ by professor Jianping Gao from Shanxi Medical University, Shanxi Province, China. The faba bean seeds (Variety: Lincan No.5) were obtained from Gansu Academy of Agricultural Sciences, Lanzhou, China. Both materials are commonly cultivated varieties in the region and were not collected from the wild.

Healthy, uniformly sized *C. pilosula* seedlings with ≥ 1 apical bud were selected for transplanting. Monoculture *C. pilosula* was planted with a row spacing of 25 cm and a plant spacing of 8 cm. The intercropping system was arranged in a 2:1 row ratio (two rows of *C. pilosula* alternating with one row of faba bean), and the space for intercropped *C. pilosula* was the same as in monoculture, while faba bean was planted with a row spacing of 50 cm and a plant spacing of 25 cm. The plot size was 24 m^2^ (3 m × 8 m). *C. pilosula* was transplanted on 25 March and harvested on 15 October, and faba bean was sowed on 6 May. No irrigation was applied during the experiment, weeds were controlled manually, and no herbicides or insecticides were used.

### Sampling and measurement

At the peaking flowering stage of *C. pilosula* (25 August), leaf traits and physiological parameters were measured. The net photosynthetic rate (Pn), stomatal conductance (Gs), transpiration rate (Tr), and intercellular CO_2_ concentration (Ci) of leaves were measured using an Li-6400 photosynthesis system (LI-COR Biosciences, USA). Six fully expanded leaves per plot from randomly selected *C. pilosula* plants were measured between 9:00 and 11:00 am under a reference CO_2_ concentration of 400 μmol mol⁻^1^. Instantaneous water use efficiency (IWUE), defined as the ratio of net photosynthetic rate to transpiration rate (Pn/Tr), was calculated to assess the trade-off between carbon gain and water loss at the leaf level. Concurrently, mature leaves were collected for morphological analysis (leaf length and width) and enzymatic activity assays (immediately frozen in liquid N). For enzyme activity assays, frozen leaf samples were ground and homogenized in specific ice-cold extraction buffers provided by the respective assay kits. The homogenates were centrifuged at 4 °C, and the supernatants were used for immediate determination. Activities of sucrose synthase (SS), sucrose phosphate synthase (SPS), nitrate reductase (NR), and glutamate synthase (GOGAT) were assayed using commercial kits from Beijing Solarbio Science & Technology Co., Ltd, while UDP-glucose pyrophosphorylase (UGPase) and phosphomannomutase (PMM) activities were assayed using kits from Shanghai Bohu Biotechnology Co., Ltd., strictly following the manufacturers’ protocols. All enzymatic activities were expressed on a fresh weight basis.

**Fig. 1:**
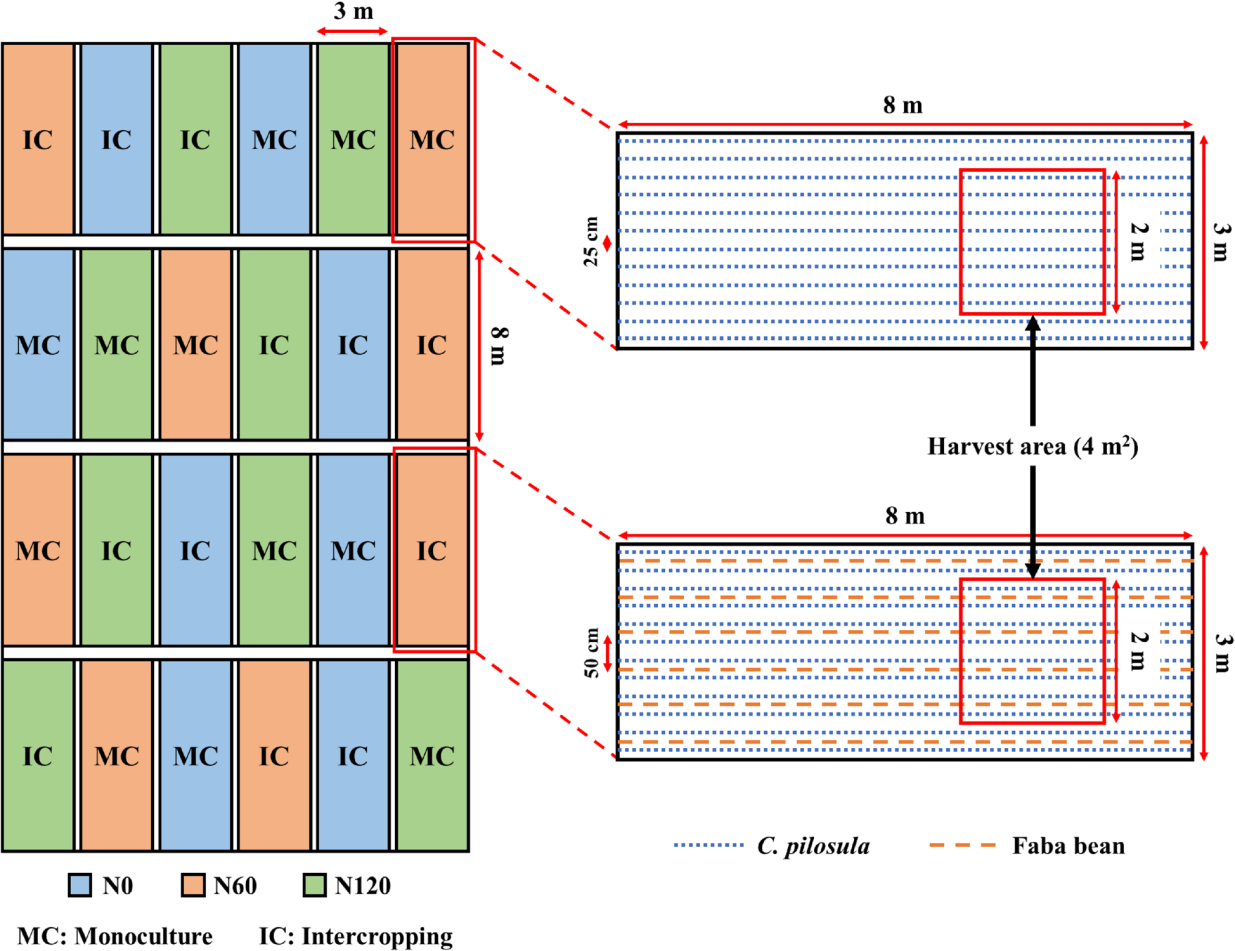
Diagrammatic representation of experimental plots and schematic diagram of monoculture and intercropping layout

To determine *C. pilosula* root yield, plants were harvested on 15 October from a central 4 m^2^ (2 m × 2 m) quadrat in each plot, excluding the outer rows to minimize border effects (Fig. 1). In monoculture plots, this quadrat contained only *C. pilosula* plants at density consistent with the overall plot. In intercropping plots, the same 4 m^2^ quadrat contained both *C. pilosula* and faba bean rows arranged in the same 2:1 row ratio as the overall plot. Only *C. pilosula* roots within the quadrat were collected, washed, and weighed, faba bean plants were excluded from measurement. Root yield was calculated on a per-unit-land-area basis using the following formula, which was applied identically to both cropping systems:

$$\begin{aligned}\mathrm{Yield}\;(\mathrm{kg}\;\mathrm{ha}^{-1})\;&=\;(\mathrm{Root}\;\mathrm{weight}\;\mathrm{from}\;\mathrm{quadrat}\;(\mathrm{kg}))\;/\;(4\;\mathrm m^2)\;\\&\times\;10000\;\mathrm m^2\;\mathrm{ha}^{-1}\end{aligned}$$

Faba bean grain yield was not measured in this study, as the primary focus was on the medicinal root yield and quality of *C. pilosula*; consequently, the land equivalent ratio (LER) could not be calculated. Six *C. pilosula* plants per plot were selected to measure root length and diameter. The roots were then oven-dried and weighed to calculate the single root dry mass. Dry roots were ground to a fine powder and used for active ingredient analysis.

The concentrations of lobetyolin, atractylenolide III, and syringin were determined using the method described by Huang et al. (2024) [4]. Briefly, 0.1 g of root powder was extracted with 25 mL of 75% methanol for 45 min at 40 ℃ using ultrasonication (50 kHz, 40 W). After centrifugation at 10,000 rpm for 15 min, the supernatant was filtered through a 0.22-μm membrane. Filtered samples were analyzed using an ACQUITY UPLC BEH C18 column (2.1 mm × 50 mm, 1.7 μm; Waters Corporation, USA). The column temperature was 30 ℃, the flow rate was 0.4 mL min^−1^, and the injection volume was 2 μL. Gradient elution was performed using a mobile phase consisting of methanol (A) and 0.1% ammonium formate solution (B) under the following conditions: 5% A at 0–0.5 min; 5–95% A at 0.5–5 min; 95% A at 5–7 min; 95%−5% A at 7–7.1 min; 5% A at 7.1–9 min. The detection wavelength was 220 nm. Standard lobetyolin, atractylenolide III, and syringin were obtained from the China National Institutes for Drug Control, with standard dilutions of 10, 50, 100, 400, 600, and 1000 ng mL^−1^. Each biological replicate sample was analyzed in duplicate (two technical replicates), and the averaged values from the two technical replicates were used in all subsequent statistical analyses.

Nitrogen partial factor productivity (NPFP) and agronomic nitrogen use efficiency (aNUE) were calculated to evaluate the efficiency of nitrogen fertilizer utilization. These indices were defined as follows:

$$\mathrm{NPFP}\;(\mathrm{kg}\;\mathrm{kg}^{-1})\;=\;\mathrm Y/{\mathrm N}_{\mathrm{fer}}$$

Where Y is the root yield of *C. pilosula* (kg ha^−1^), and N_fer_ is the N fertilizer application rate (kg N ha^−1^). NPFP reflects the overall productivity per unit of N input.

$$\mathrm{aNUE}\;=\;({\mathrm Y}_{\mathrm N-}{\mathrm Y}_0)/{\mathrm N}_{\mathrm{fer}}$$

where Y_N_ is the root yield (kg ha^−1^) in N fertilizer treatment, Y_0_ is the root yield (kg ha^−1^) in no N fertilizer treatment, and N_fer_ is the N fertilizer application rate (kg N ha^−1^). aNUE represents the incremental yield gain from N fertilization.

### Statistical analysis

Two-way analysis of variance (ANOVA) was conducted to evaluate the effects of N level, cropping system, and their interaction on root properties, photosynthetic parameters, enzyme activity, and N use efficiency. Cropping system, N level, and their interaction were treated as fixed factor, and Block was treated as a random factor. Multiple comparisons were performed using the Tukey’s HSD test at *P* < 0.05. Mantel tests were conducted to quantify and test the significance of correlations between root properties and leaf trait matrices, and principal component analysis (PCA) was performed to further visualize how these relationships were structured by cropping system and N application level. These analyses were conducted using individual plot-level data (*n* = 24), and all variables were standardized using Z-score method. All statistical analyses were conducted using R version 4.1.3 [20].

## Results

### Effects of N level and cropping system on root growth and yield of *C. pilosula*

Cropping system had a significant effect (*P* < 0.05) on root length, root diameter, and single-root dry mass, whereas N level and the interaction effect did not significantly influence these root traits (Table 1). Under N0 and N60 treatments, intercropped *C. pilosula* exhibited significantly greater root length, root diameter, and single-root dry mass compared to monoculture. No significant differences were observed between monoculture and intercropping under the N120 treatment. Root trait values in monocultured *C. pilosula* peaked at the highest N level, whereas intercropped plant showed maximal root trait values under moderate N condition.

**Table 1:** Effects of N level and cropping system on root growth and yield of *C. pilosula* at harvest season

N level	Cropping system	Root length (cm)	Root diameter (mm)	Root dry mass (g plant^−1^)	Yield (kg hm^−2^)
N0	MC	24.74 ± 0.65c	7.23 ± 0.51c	3.03 ± 0.04d	909.05 ± 10.57d
	IC	26.99 ± 0.67ab	7.80 ± 0.43bc	3.20 ± 0.07bc	990.23 ± 20.61b
N60	MC	25.28 ± 0.32c	7.85 ± 0.23bc	3.10 ± 0.05 cd	932.36 ± 15.64 cd
	IC	28.36 ± 0.44a	10.01 ± 0.66a	3.38 ± 0.06a	1106.28 ± 20.84a
N120	MC	26.11 ± 0.40bc	7.93 ± 0.66bc	3.20 ± 0.05bcd	963.20 ± 15.78bc
	IC	27.08 ± 0.05ab	8.96 ± 0.29ab	3.28 ± 0.03ab	994.23 ± 9.63b
Variation source					
N level (N)		NS	NS	NS	**
Cropping system (CS)		***	*	**	***
N × CS		NS	NS	NS	**

MC represent *C. pilosula* monoculture, IC represent *C. pilosula* intercropping. Data in the table are the means ± standard error. Different letters indicate significant differences among different treatments (Tukey HSD). NS, no significant

^*^P < 0.05

^**^P < 0.01

^***^P < 0.001

Nitrogen level, cropping system, and their interaction all had highly significant effects on root yield (Table 1). Compared with monoculture, intercropped *C. pilosula* increased root yield by 8.9% under N0 level and by 18.7% under N60. No significant difference was found between monoculture and intercropping under N120. Monocultured *C. pilosula* yield increased steadily with N application, and N60 and N120 promoting yield by 2.6% and 6.0%, respectively, compared to no N application. Intercropped *C. pilosula* achieved the highest root yield at N60 level.

### Effects of N level and cropping system on active ingredient content of *C. pilosula*

N level and cropping system had significant effects on the concentrations of lobetyolin, atractylenolide III, and syringin (Table 2). At the same N level, intercropping increased the concentration of lobetyolin by 5.5–30.0%, atractylenolide III by 8.1–56.9%, and syringin by 12.4–29.4%, respectively, compared with monoculture. The effects of N level on active ingredient concentration depended on the cropping system. Monocultured *C. pilosula* exhibited the highest concentrations of lobetyolin and atractylenolide III at N60 level, while syringin concentration decreased with increasing N fertilization. In intercropped *C. pilosula*, the concentrations of all three compounds declined with increasing N fertilizer.

**Table 2:** Effects of N level and cropping system on active ingredient concentration and yield of *C. pilosula*

N level	Cropping system	Active ingredient concentration			Active ingredient yield		
		Lobetyolin (mg g^−1^ dry mass)	Atractylenolide III (mg g^−1^ dry mass)	Syringin (mg g^−1^ dry mass)	Lobetyolin (g hm^−2^)	Atractylenolide Ⅲ (g hm^−2^)	Syringin (g hm^−2^)
N0	MC	0.90 ± 0.01c	0.03 ± 0.00c	0.03 ± 0.00b	817.99 ± 8.34d	29.99 ± 4.08b	28.14 ± 1.12b
	IC	1.08 ± 0.05a	0.05 ± 0.00a	0.04 ± 0.00a	1070.96 ± 30.54a	51.52 ± 5.22a	34.67 ± 0.21a
N60	MC	0.96 ± 0.05bc	0.04 ± 0.00bc	0.03 ± 0.00c	897.10 ± 39.92c	33.06 ± 4.21b	23.49 ± 1.23c
	IC	1.02 ± 0.05ab	0.05 ± 0.00ab	0.03 ± 0.00ab	1123.11 ± 43.61a	50.27 ± 3.40a	36.03 ± 1.99a
N120	MC	0.77 ± 0.03d	0.03 ± 0.00c	0.02 ± 0.00c	738.25 ± 27.52e	25.46 ± 0.50b	21.50 ± 1.06c
	IC	0.99 ± 0.01b	0.03 ± 0.00c	0.03 ± 0.00c	987.78 ± 21.96b	28.42 ± 1.79b	25.02 ± 1.98bc
Variation source							
N level (N)		**	**	***	***	**	***
Cropping system (CS)		***	**	***	***	***	***
N × CS		*	NS	NS	NS	*	*

MC represent *C. pilosula* monoculture, IC represent *C. pilosula* intercropping. Data in the table are the means ± standard error. Different letters indicate significant differences among different treatments (Tukey HSD). NS, no significant

^*^, *P* < 0.05

^**^, *P* < 0.01

^***^, *P* < 0.001

Active ingredients yields were calculated by multiplying concentration by root yield and were significantly affected by N level, cropping system, and their interaction (Table 2). On average, lobetyolin, atractylenolide III, and syringin yields in intercropped *C. pilosula* were 29.7%, 47.1%, and 30.9% higher than in monoculture, respectively. Distinct response patterns to N fertilization were observed among the three compounds. Lobetyolin yield was highest at the N60 treatment in both cropping systems. N level had no significant effect on atractylenolide III yield in monoculture, but higher N fertilization decreased atractylenolide III yield in the intercropping system. N fertilization decreased syringin yield in monoculture, whereas only excessive N fertilization decreased syringin yield in the intercropping system.

### Effects of N level and cropping system on leaf size and photosynthetic parameters

N level and cropping system significantly altered leaf length and width (Table 3). Across N levels, intercropping significantly increased leaf length and width by 8.4% and 5.2%, respectively, compared to monoculture. In monoculture, leaf length and width were not affected by N level, whereas in intercropping systems, the N60 treatment was associated with the largest leaf length and width.

**Table 3:** Effects of N level and cropping system on leaf size and photosynthetic parameters of *C. pilosula*

N level	Cropping system	Leaf length (cm)	Leaf width (cm)	Pn (μmol m^−2^ s^−1^)	Gs (mol m^−2^ s^−1^)	Tr (mmol m^−2^ s^−1^)	Ci (μmol mol^−1^)	IWUE (μmol mol^−1^)
N0	MC	3.56 ± 0.08c	2.55 ± 0.03c	13.33 ± 0.64c	0.19 ± 0.01ab	3.34 ± 0.33d	222.92 ± 5.37d	4.04 ± 0.30a
	IC	3.93 ± 0.03b	2.66 ± 0.06bc	15.09 ± 0.42b	0.22 ± 0.01a	4.45 ± 0.15b	252.94 ± 4.07bc	3.39 ± 0.03bc
N60	MC	3.71 ± 0.10bc	2.65 ± 0.06bc	13.42 ± 0.51bc	0.22 ± 0.01a	3.77 ± 0.08 cd	261.23 ± 4.96b	3.57 ± 0.18b
	IC	4.23 ± 0.07a	2.89 ± 0.07a	17.21 ± 0.35a	0.23 ± 0.02a	5.62 ± 0.11a	291.79 ± 4.43a	3.06 ± 0.02c
N120	MC	3.81 ± 0.07bc	2.72 ± 0.06abc	14.12 ± 0.44bc	0.16 ± 0.01b	3.96 ± 0.11bc	243.42 ± 6.13c	3.57 ± 0.06b
	IC	3.85 ± 0.11b	2.77 ± 0.04ab	14.59 ± 0.68bc	0.19 ± 0.01ab	4.15 ± 0.21bc	263.79 ± 1.72b	3.52 ± 0.05b
Variation source								
N level (N)		NS	*	NS	*	**	***	NS
Cropping system (CS)		***	*	**	*	***	***	**
N × CS		*	NS	*	NS	**	NS	NS

Data in the table are the means ± standard error. Different letters indicate significant differences among different treatments (Tukey HSD). NS, no significant

MC represent *C. pilosula* monoculture, IC represent *C. pilosula* intercropping. Pn, net photosynthetic rate

*Gs* stomatal conductance, *Tr* Transpiration rate, *Ci* Intercellular CO_2_ concentration, *IWUE* Iinstantaneous water use efficiency

^*^, *P* < 0.05

^**^, *P* < 0.01

^***^, *P* < 0.001

N level and cropping system significantly affected stomatal conductance (Gs), transpiration rate (Tr), and intercellular CO₂ concentration (Ci). Net photosynthetic rate (Pn) and instantaneous water use efficiency (IWUE) were significantly affected by cropping system (Table 3). Under no N and moderate N levels, leaf Pn, Tr, and Ci in the intercropping system were significantly higher than in monoculture, while IWUE was significantly lower. No significant differences were observed between cropping systems (except Ci). Photosynthetic parameters showed quadratic responses to N level, peaking at the N60 treatment. Intercropping combined with the N60 treatment was associated with the highest Pn, Gs, Tr, and Ci.

### Effects of N level and cropping system on leaf enzyme activity

N level and cropping system had significant effects on leaf C and N metabolic enzyme activities, with no significant interaction observed between N level and cropping system (Table 4). Compared with monoculture, intercropping increased NR activity by 9.3–61.9%, GOGAT activity by 9.3–61.6%, SS activity by 4.3–18.6%, SPS activity by 9.3–61.6%, UGPase activity by 9.9–67.2%, and PMM activity by 10.4–28.1%. Nitrogen fertilization significantly increased NR and GOGAT activities in monocultured *C. pilosula* but did not affect these activities in intercropped plants. In contrast, N fertilization increased the activity of SS, SPS, UGPase, and PMM in both monocultured and intercropped *C. pilosula*.

**Table 4:** Effects of N level and cropping system on leaf C and N metabolic enzyme activity of *C. pilosula*

N level	Cropping system	NR (mg g^−1^ h^−1^)	GOGAT (mg g^−1^ h^−1^)	SS (mg g^−1^ h^−1^)	SPS (mg g^−1^ h^−1^)	UGPase (mg g^−1^ h^−1^)	PMM (mg g^−1^ h^−1^)
N0	MC	17.72 ± 1.43c	5.91 ± 0.48c	34.75 ± 0.29c	5.91 ± 0.48c	5.51 ± 0.71d	56.51 ± 2.58d
	IC	28.69 ± 1.91ab	9.56 ± 0.63ab	37.12 ± 2.46c	9.56 ± 0.63ab	9.21 ± 0.86c	68.35 ± 1.46bc
N60	MC	25.53 ± 1.64b	8.51 ± 0.54b	46.71 ± 0.30b	8.51 ± 0.54b	15.35 ± 0.83b	60.49 ± 4.28 cd
	IC	27.91 ± 2.92ab	9.30 ± 0.97ab	55.41 ± 2.69a	9.30 ± 0.97ab	16.87 ± 0.83b	77.48 ± 2.36a
N120	MC	25.53 ± 1.64b	8.51 ± 0.54b	49.39 ± 1.02b	8.51 ± 0.54b	16.63 ± 1.57b	71.13 ± 1.66ab
	IC	33.37 ± 1.69a	11.15 ± 0.56a	51.50 ± 1.30ab	11.05 ± 0.56a	23.28 ± 0.49a	78.50 ± 2.92a
Variation source							
N level (N)		*	*	***	*	***	**
Cropping system (CS)		**	**	*	**	***	***
N × CS		NS	NS	NS	NS	NS	NS

MC represent *C. pilosula* monoculture, IC represent *C. pilosula* intercropping

Data in the table are the means ± standard error. Different letters indicate significant differences among different treatments (Tukey HSD). NS, no significant

^*^, *P* < 0.05

^**^, *P* < 0.01

^***^, *P* < 0.001

### Effects of N level and cropping system on N use efficiency

N level, cropping system, and their interaction significantly influenced N partial factor productivity and agronomic N use efficiency (Table 5). At the N60 level, intercropping significantly increased N partial factor productivity by 18.7% and agronomic N use efficiency by 394.9% compared to monoculture. No significant effect was observed between monoculture and intercropping at the N120 level. The N120 treatment significantly decreased N partial factor productivity and agronomic N use efficiency compared to N60 treatment.

**Table 5:** Effect of N level and cropping system on N use efficiency of *C. pilosula*

N level	Cropping system	N partial factor productivity	Agronomic N use efficiency
N60	MC	15.54 ± 0.26b	0.39 ± 0.25b
	IC	18.44 ± 0.35a	1.93 ± 0.20a
N120	MC	8.03 ± 0.13c	0.45 ± 0.17b
	IC	8.29 ± 0.08c	0.03 ± 0.25b
Variation source			
N level (N)		***	**
Cropping system (CS)		***	*
N × CS		**	**

MC represent *C. pilosula* monoculture, IC represent *C. pilosula* intercropping

Data in the table are the means ± standard error. Different letters indicate significant differences among different treatments (Tukey HSD). NS, no significant

^*^, *P* < 0.05

^**^, *P* < 0.01

^***^, *P* < 0.001

### Relationships among root yield, active ingredient content, and leaf parameters

**Fig. 2:**
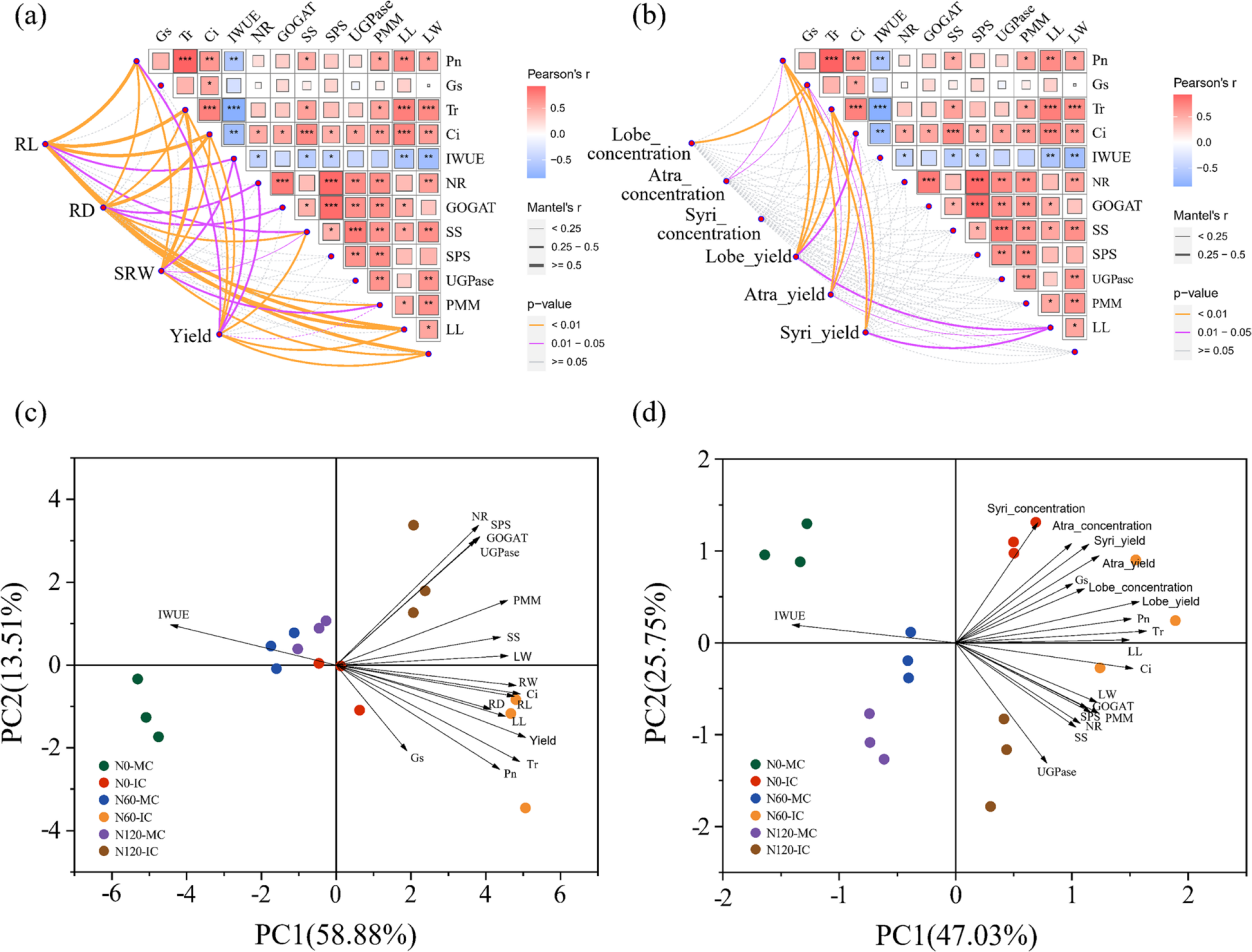
**a**, **b** Relationships among root yield, active ingredient content, and leaf parameters of *C. pilosula* based on Mantel test. **c**, **d** Principal Component Analysis of among root yield, active ingredient content, and leaf parameters of *C. pilosula*. LL, leaf length (cm); LW, leaf width (cm); NR, nitrate reductase (mg g^−1^ h^−1^); GOGAT, glutamate synthase (mg g^−1^ h^−1^); SS, sucrose synthase (mg g^−1^ h^−1^); SPS, sucrose phosphate synthase (mg g^−1^ h^−1^); UGPase, UDP-glucose pyrophosphorylase (mg g^−1^ h^−1^); PMM, phosphomannomutase (mg g^−1^ h^−1^); Pn, net photosynthetic rate (μmol m^−2^ s^−1^); Gs, stomatal conductance (mol m^−2^ s^−1^); Tr, transpiration rate (mmol m^−2^ s^−1^); Ci, intercellular CO_2_ concentration (μmol mol^−1^); IWUE, instantaneous water use efficiency (μmol mol^−1^); RL, root length (cm); RD, root diameter (mm); RW, root dry mass (g plant^−1^); Lobe_concentration, lobetyolin concentration (mg g^−1^ dry mass); Atra_concentration, atractylenolide III concentration (mg g^−1^ dry mass); Syri_concentration, syringin concentration (mg g^−1^ dry mass); Lobe_yield, lobetyolin yield (g hm^−2^); Atra_ yield, atractylenolide III yield (g hm^−2^); Syri_ yield, syringin yield (g hm^−^.^2^)

Mantel test revealed that root traits and yield were significantly correlated with leaf net photosynthetic rate, transpiration rate, sucrose synthase activity, leaf length, and leaf width (Fig. 2a). Lobetyolin and atractylenolide III concentration were correlated with leaf net photosynthetic rate and stomatal conductance, while lobetyolin, atractylenolide III, and syringin yields were significantly correlated with leaf photosynthetic parameters and size (Fig. 2b).

Principal component analysis (PCA) was used to evaluate how root yield, active ingredient content, and leaf traits were associated with N levels and cropping systems. For the encompassing root yield and leaf traits (Fig. 2c), the first two principal components explained 72.4% of the total variance, with PC1 accounting for 58.9%. PC1 exhibited strong positive loadings for root yield, leaf length and width, Pn, and Gs, while IWUE showed a strong negative loading on this component. Notably, cropping systems were clearly separated along PC1, with intercropped samples clustering on the positive side and monocultured samples on the negative side. Nitrogen application levels showed no clear separation along either PC1 or PC2. For the analysis of active ingredient content and leaf traits (Fig. 2d), the first two principal components together accounted for 72.8% of the total variance. PC1 (47% of variance) showed strong positive loadings on leaf length, Pn, Tr, and lobetyolin yield, while PC2 (25.8% of variance) showed strong positive loadings on syringin and atractylenolide III concentrations. Cropping systems were clearly separated along PC1, with intercropped samples positioned on the positive side. In contrast, N application levels were separated along PC2, with low N (N0) samples clustering on the positive side and high N (N120) samples on the negative side.

## Discussion

### Intercropping with faba bean increased root yield and active ingredient content of *C. pilosula*

Our study found that intercropping with faba bean promoted root growth of *C. pilosula* and increased root yield by 10.3% (Table 1). These findings are consistent with our previous study [4]. Ecologists and agronomists have demonstrated that interspecific interaction in certain plant communities can improve productivity through niche partitioning and resource complementarity [7]. This phenomenon has been confirmed in various medicinal-based intercropping systems. For example, intercropping with *Allium fistulosum* increased the root yield of *Platycodon grandiflorus* by regulating interaction among “plant physiology and biochemistry-root exudates-rhizosphere microflora” [9]. Similarly, intercropping with legumes (field bean and cowpea) enhanced patchouli yield and economic returns through improved resource use efficiency [6]. Intercropping with maize increased rhizome yield of *Atractylodes lancea* by altering nutrient availability, microbial community structure, and volatile organic compounds in the rhizosphere soil [5, 11, 21]. The observed yield advantages in these medicinal plant intercropping systems are primarily attributed to interspecific facilitation being significantly greater than interspecific competition.

According to our results, intercropping with faba bean improved both the concentration and yield of active ingredients in *C. pilosula* roots (Table 2). Intercropping has been shown to increase the active ingredient content of medicinal plants. For example, intercropping with maize resulting in increased atractylon and atractylodin content in *Atractylodes lancea* [11, 21]. Similar studies have reported that intercropping with specific crops under appropriate planting arrangements improved the flavonoid and total phenol contents of *Platycodon grandifloras* [9, 12]. Our previous studies also demonstrated that appropriate row configurations with faba bean could improve the concentrations of actractylenolide III and syringin in *C. pilosula* [4]. However, the biosynthesis and accumulation of active ingredients can vary with seasonal environmental conditions, multi-year trials are needed to confirm the stability of these intercropping benefits for *C. pilosula* performance.

### Changes in leaf traits associated with increased root yield and active ingredient content of *C. pilosula*

Compared with monoculture, intercropping significantly increased leaf length and width of *C. pilosula* across N levels (Table 3), resulting in greater leaf area – a key determinant of light interception capacity [15, 22]. The positive correlation between leaf dimensions and root yield suggests that enhanced light capture contributed to the observed yield advantages. This morphological response likely reflects the modified microclimate in intercropping systems. Faba bean plants create partial shading and alter the light spectrum, potentially triggering shade-avoidance responses that promote leaf expansion [23].

Beyond morphology, intercropping significantly enhanced leaf photosynthetic rate (Pn), stomatal conductance (Gs), transpiration rate (Tr), and intercellular CO_2_ concentration (Ci) of *C. pilosula* (Table 3). This photosynthetic enhancement can be attributed to two complementary mechanisms. First, faba bean plants modify the within-canopy humidity. This modification promotes stomatal opening and reducing CO₂ diffusion resistance [24, 25]. Second, the physical support provided by faba bean stems allows *C. pilosula* to adopt a more favorable canopy architecture, improving light distribution across leaf strata [23, 26].

However, we simultaneously observed that intercropping reduced the instantaneous water use efficiency (IWUE) of *C. pilosula* leaves, suggesting that the photosynthetic gains may have been accompanied by increased water loss. This observed pattern – higher carbon gain with lower water use efficiency – could reflect a "water-for-carbon" strategy, in which plants may prioritize carbon acquisition over water conservation under favorable microclimatic conditions [27, 28]. The ecological significance of this strategy lies in its contribution to maximizing carbon gain during the critical growth period, ultimately supporting higher biomass accumulation in sink organs. However, we note that direct measurements of whole-plant water balance and carbon allocation would be required to definitively confirm this trade-off, and our leaf-level data are consistent with, but do not prove, such a strategy.

Our enzyme activity assays revealed that intercropping significantly upregulated key carbon metabolism enzymes in leaves, including SS, SPS, UGPase, and PMM (Table 4). These enzymes play complementary roles in carbon partitioning. SS catalyzes sucrose cleavage, providing substrates for energy metabolism and biosynthesis. SPS regulates sucrose synthesis and export. UGPase is involved in UDP-glucose production for cell wall and polysaccharide synthesis. PMM participates in GDP-mannose production for ascorbate and cell wall metabolism [29]. The coordinated upregulation of these enzymes suggests that intercropping may enhance both carbon assimilation and the subsequent processing and export of photoassimilates from source leaves to sink roots. The strong positive correlation between SS activity and root yield (Fig. 2) supports this interpretation. This mechanism is particularly relevant for *C. pilosula*, whose roots accumulate large quantities of polysaccharides as storage compounds and bioactive constituents. Enhanced SS activity likely facilitates the conversion of transported sucrose into UDP-glucose and other precursors required for polysaccharide biosynthesis in roots [29].

Intercropping significantly enhanced nitrogen assimilation enzyme activities, including NR and GOGAT (Table 4). NR catalyzes the rate-limiting step in nitrate assimilation, while GOGAT incorporates ammonium into glutamate—a central amino group donor for the biosynthesis of nitrogen-containing compounds [30, 31]. The enhanced nitrogen assimilation capacity, likely facilitated by biological N_2_ fixation from faba bean [8, 16, 32], provides the amino acid precursors required for the synthesis of secondary metabolites derived from the phenylpropanoid pathway, such as lobetyolin (a polyacetylene glycoside) and syringin (a phenylpropanoid glycoside). The biosynthesis of these compounds draws on phenylalanine (derived from the shikimate pathway) and UDP-glucose for glycosylation steps. Simultaneously, the upregulated carbon metabolism enzymes (SS, SPS, UGPase) supply the carbon skeletons and energy (ATP, UDP-glucose) required for secondary metabolic pathways. In contrast, atractylenolide III is a sesquiterpene lactone synthesized via the mevalonate pathway, which utilizes acetyl-CoA derived from carbohydrate metabolism and does not involve nitrogen-containing intermediates. Therefore, its accumulation under intercropping is more directly linked to enhanced carbon assimilation rather than nitrogen status. The coordinated enhancement of both carbon under intercropping likely contributes to the increased accumulation of all three bioactive compounds, while the influence of nitrogen status varies according to each compound’s biosynthetic requirements.

Additionally, intercropping may alter the internal C:N balance in *C. pilosula*. Under a higher C:N ratio, more resources may be allocated to the synthesis of secondary metabolites, which primarily serves defense and storage functions [33]. These shifts may enhance the concentration of key pharmacologically active ingredients, such as lobetyolin, atractylenolide III, and syringin. However, the accumulation of different active ingredients is likely regulated by distinct metabolic pathways. Future research employing transcriptomic and metabolomic analyses could precisely elucidate the regulation of biosynthetic pathways underlying the accumulation of lobetyolin, atractylenolide III, and syringin in response to intercropping.

### Yield advantage of intercropping was affected by N application rates

This study found that an appropriate N application rate (60 kg ha^−1^) increased root yield of *C. pilosula*, whereas a high N application rate (120 kg ha^−1^) decreased it. This finding indicates a clear threshold for N fertilization in optimizing root yield. Appropriate N application promotes leaf expansion and chlorophyll synthesis, thereby enhancing photosynthetic C assimilation and carbohydrate production. This facilitates the translocation of assimilates to the roots, ultimately increasing root biomass. In contrast, excessive N induces a substantial C sink demand for N assimilation, leading to carbohydrate depletion [34]. Concurrently, excessive N application stimulates rapid aboveground growth, reduces the root-to-shoot ratio, and significantly diminishes the proportion of dry matter allocated to the root, consequently suppressing root yield.

Our study showed that appropriate N application increased the yield advantages of intercropping *C. pilosula*, while excessive N application decreased these advantages. This indicates that interspecific interaction in *C. pilosula*/faba bean intercropping systems are affected by N fertilization. Interspecific N complementarity through N_2_ fixation by legumes is a widespread mechanism for increase yield in intercropping systems. Intercropping with non-legumes enhanced symbiotic N_2_ fixation of legumes under low to moderate soil N conditions. Nitrogen fixed by legumes could transfer to non-legumes, promoted their growth [16]. In contrast, high soil N content inhibits symbiotic N_2_ fixation in legumes, weakening interspecific N facilitation. Moreover, excessive N stimulates rapid aboveground growth, intensifying light competition between *C. pilosula* and faba bean and thereby inhibiting the growth of *C. pilosula* [35].

From a practical perspective, the optimal N rate of 60 kg ha⁻^1^ not only maximizes yield and quality but also reduces N fertilizer input by 50% compared to conventional practices (120 kg ha⁻^1^) under the conditions tested. While these results are promising, further validation across multiple growing seasons and locations, as well as direct measurements of environmental impacts (e.g. N leaching, greenhouse gas emissions), would be needed to fully assess the sustainability benefits of this intercropping system.

## Conclusions

Our study demonstrates that intercropping with faba bean increases root yield and active ingredient content of *C. pilosula*. These improvements are associated with enhanced leaf photosynthesis, upregulated sucrose synthase and nitrate reductase activities, and a physiological pattern consistent with prioritized carbon gain over water conservation (i.e., reduced water use efficiency accompanied by higher photosynthetic rates). The magnitude of intercropping advantages for *C. pilosula* was dependent on N fertilizer level, with the optimal rate (60 kg N ha^−1^) strengthening them, while excessive N (120 kg N ha^−1^) diminished them under the conditions tested. These findings suggest that optimizing N fertilization in *C. pilosula*/faba bean intercropping system may represent a promising strategy for promoting more ecological cultivation of *C. pilosula*, though further multi-year and multi-location trials are needed to confirm the generalizability of these results.

## Data Availability

The datasets used during the current study are available from the corresponding author on reasonable request.
